# Proteome analysis of urinary biomarkers in a cigarette smoke-induced COPD rat model

**DOI:** 10.1186/s12931-022-02070-1

**Published:** 2022-06-15

**Authors:** Weiwei Qin, He Huang, Yuting Dai, Wei Han, Youhe Gao

**Affiliations:** 1 Department of Anesthesiology, Qingdao Municipal Hospital, Qingdao University, Qingdao, 266071 China; 2 Department of Biochemistry and Molecular Biology, Gene Engineering Drug and Biotechnology Beijing Key Laboratory, Beijing Normal University, Beijing, 100875 China; 3 Department of Respiratory Medicine, Qingdao Municipal Hospital, Qingdao University, Qingdao, 266071 China

**Keywords:** Urinary proteome, Biomarker, Cigarette smoking, COPD, Rat model, LC–MS/MS

## Abstract

**Background:**

Chronic obstructive pulmonary disease (COPD) is a chronic inflammatory airway disease caused by inhalation of cigarette smoke (CS) and other harmful gases and particles.

**Methods:**

This study aimed to explore potential urinary biomarkers for CS-induced COPD based on LC–MS/MS analysis.

**Results:**

A total of 340 urinary proteins were identified, of which 79 were significantly changed (30, 31, and 37 at week 2, 4 and 8, respectively). GO annotation of the differential urinary proteins revealed that acute-phase response, response to organic cyclic compounds, complement activation classical pathway, and response to lead ion were significantly enriched at week 2 and 4. Another four processes were only enriched at week 8, namely response to oxidative stress, positive regulation of cell proliferation, thyroid hormone generation, and positive regulation of apoptotic process. The PPI network indicated that these differential proteins were biologically connected in CS-exposed rats. Of the 79 differential proteins in CS-exposed rats, 56 had human orthologs. Seven proteins that had changed at week 2 and 4 when there were no changes of pulmonary function and pathological morphology were verified as potential biomarkers for early screening of CS-induced COPD by proteomic analysis. Another six proteins that changed at week 8 when obvious airflow obstruction was detected were verified as potential biomarkers for prognostic assessment of CS-induced COPD.

**Conclusions:**

These results reveal that the urinary proteome could sensitively reflect pathological changes in CS-exposed rats, and provide valuable clues for exploring COPD biomarkers.

**Supplementary Information:**

The online version contains supplementary material available at 10.1186/s12931-022-02070-1.

## Background

Chronic obstructive pulmonary disease (COPD) is a common, preventable, and treatable disease characterized by persistent respiratory symptoms and airflow limitation. It is an important public health challenge, and is now the third leading cause of death worldwide [1]. COPD is projected to continue to contribute to an increase in the overall worldwide burden of disease in the coming decades [2]. The airway and/or alveolar abnormalities were usually caused by exposure to cigarette smoking (CS) and other noxious gases or particles. The main mechanisms underlying COPD include amplified inflammation, oxidative stress, protease-antiprotease imbalance, and peribronchiolar and interstitial fibrosis [3, 4].

Despite increasing knowledge regarding COPD pathophysiology, substantial gaps remain regarding diagnosis and, in particular, early detection. Spirometry is by far the primary diagnostic approach, according to the criteria provided by the Global Initiative for Chronic Obstructive Lung Disease (GOLD), the American Thoracic Society (ATS), the European Respiratory Society (ERS), and the Japanese Respiratory Society (JRS). However, it cannot be reliably used as the only diagnostic test because of its weak specificity, and it is not recommended for the evaluation of airflow limitations when testing respiratory function in patients without respiratory symptoms [5]. Mounting evidence suggests that COPD is either underdiagnosed or misdiagnosed in approximately two-thirds of patients at risk of COPD [6]. Early COPD diagnosis has remained challenging due to the small impact of early lung function loss. In this study, we intend to explore new non-invasive biomarkers for the diagnosis of early COPD to enable timely and accurate interventions.

There is growing awareness of the need to identify new non-invasive biomarkers for the early screening and detection of COPD. In recent decades, mass spectrometry (MS)-based proteomics has dramatically improved and emerged as an important tool for identifying biomarkers. Several potential biomarkers of COPD have been described and categorized as primarily blood and sputum biomarkers [7, 8]. Some of these candidate blood protein biomarkers include C-reactive protein (CRP), fibrinogen, surfactant protein D, club cell protein 16, brain natriuretic peptide, soluble receptor for advanced glycation end-products and immunoglobulins [9], and lipocalins, matrix metalloproteinases, several inflammatory cytokines, and polymeric immunoglobulin receptor are some of the promising sputum biomarkers [8]. However, none of these candidate biomarkers could be successfully translated clinically. Shotgun proteomics of blood and sputum have been largely disappointing as the discovered protein biomarkers have lacked resolution or specificity to the condition.

Urine can be both sampled noninvasively and continuously. Moreover, compared with blood, urine proteome may reflect changes in disease progression at the early stage, for lack of mechanisms for maintaining homeostasis. Urinary proteomic studies have discovered several candidate biomarkers for pulmonary diseases, such as lung cancer [10], pulmonary fibrosis [11], and ventilation-induced lung injury [12]. The urinary proteome showed obvious changes even in the absence of clinical manifestations or histopathological damage to lung tissue, as in the bleomycin-induced pulmonary fibrosis rat model [11] and the ovalbumin (OVA)-induced asthma mouse model [13]. Therefore, the urinary proteome might sensitively reflect pathophysiological changes in the lung at an early stage and is a promising resource for studying the biomarkers of pulmonary diseases.

In this study, we establish a rat model of short-term CS exposure to simulate the pathogenesis of human early COPD [14]. We intend to explore potential urinary protein biomarkers to screen for early COPD based on proteomics technology.

## Materials and methods

### Animals

Male Wistar’s rats (weight range: 180–200 g; 8 weeks of age) were purchased from Beijing Vital River Laboratory Animal Technology Co., Ltd. The rats were acclimatized for 1 week before the experiment. The animal experiments were reviewed and approved by Qingdao Municipal Hospital Medical Ethics Committee. All methods were carried out in accordance with relevant guidelines and regulations of the National Health Commission and the Ministry of Science and Technology and performed in accordance with the guidelines for animal research.

### Model establishment

The rats with COPD-like lung disease were established by the CS method. The rats were randomly divided into a control group (room air-exposed, n = 18) and a CS group (CS-exposed, n = 18). Commercial non-filtered cigarettes (trade name: DA QIAN MEN) containing 11 mg tar and 0.8 mg nicotine per cigarette were used in this study. In detail, three rats were kept in a chamber with size of 36 cm (length) × 20 cm (width) × 28 cm (height) and exposed to successive periods of CS at a rate of approximately 10 min per cigarette. At intervals of 1 min, the smoke of a new cigarette was delivered into the chamber and 6 cigarettes for 1 h in the morning and 6 cigarettes 1 h in the afternoon for 6 days per week. After exposure, the rats were returned to their cages. Control animals (sham group) inhaled clean (filtered) air only. All of the rats were maintained throughout the study in specific-pathogen-free conditions ventilated with clean air at 20–25 °C. The lights were on a 12-h cycle. Water and diets were provided ad libitum, excluding the CS exposure period.

### Pulmonary function test and lung histopathology

Pulmonary function was evaluated by the AniRes2005 animal lung function analysis system (Beijing Beilanbo Technology). The forced vital capacity (FVC) and forced expiratory volume in 0.3 s (FEV0.3), expiratory resistance (RE), and dynamic lung compliance (Cdy) were measured, and the ration of FEV0.3/FVC was calculated.

The lung was harvested at week 2, 4 and 8 and fixed in 4% paraformaldehyde for 24 h. The fixed tissues were embedded in paraffin, sectioned at 4 μm and stained with hematoxylin and eosin (HE) and alcian blue-periodic acid-Schiff (AB-PAS) to reveal histopathological lesions.

### Urine collection and sample preparation

Urine samples from the COPD rat model induced by smoking were taken at week 2, 4 and 8. After collection, the urine was centrifuged at 4 °C for 30 min at 3000×*g* and then at 12,000×*g* to remove pellets. Three volumes of ethanol (− 20 °C precooling) were added to the supernatant, which was shaken well and then precipitated in a – 20 °C refrigerator overnight. The next day, the urine was centrifuged at 4 °C at 12,000×*g* for 30 min, and the supernatant was discarded. The pellet was then resuspended in lysis buffer (8 M urea, 2 M thiourea, 50 mM Tris, and 25 mM DTT). The protein concentrations were measured using the Bradford method. Proteins were digested with trypsin (Trypsin Gold, 122 Mass Spec Grade, Promega, Fitchburg, Wisconsin, USA) using filter-aided sample 123 preparation methods [15]. The peptide mixtures were desalted using Oasis HLB cartridges (Waters, Milford, MA) and dried by vacuum evaporation.

### Liquid chromatography coupled with tandem mass spectrometry (LC–MS/MS) analysis

The digested peptides were acidified with 0.1% formic acid and then loaded onto a reversed-phase micro-capillary column using the Thermo EASY-nLC 1200 HPLC system. The MS data were acquired using the Thermo Orbitrap Fusion Lumos (Thermo Fisher Scientific, Bremen, Germany). The elution gradient for the analytical column was 95% mobile phase A (0.1% formic acid; 99.9% water) to 40% mobile phase B (0.1% formic acid; 89.9% acetonitrile) over 60 min at a flow rate of 300 nL/min.

### Label-free proteome quantification

The LC–MS/MS results were analyzed using Mascot software and Progenesis software. The database used was the SwissProt_Rat database (8091 sequences). The search conditions were trypsin digestion, fixed modification: carbamidomethylation of cysteines, variable modification: oxidation of ethionine, and the tolerances of the parent ion and fragment ion were both 0.02 Da. After normalization, the mass spectrometry peak intensity was used to analyze differential proteins between the control and CS groups.

### Bioinformatic analysis

GO analysis was performed on the 79 differential urinary proteins identified in CS-induced COPD rat model (http://www.geneontology.org/) [16, 17]. In this study, significant GO enrichment was defined at p < 0.05. STRING database (http://www.string-db.org) was used to construct protein–protein interaction (PPI) networks. The database of known and predicted protein interactions, including direct (physical) and indirect (functional) associations.

The ‘Wu Kong’ platform (https://www.omicsolution.org/wkomics/main/) was used for statistical analysis. The differential proteins were selected using one-way ANOVA, and p-values were adjusted using the Benjamini–Hochberg method. Significance was set at a fold change of 1.5 and a p-value of < 0.05.

## Results

### Characterization of CS-induced COPD in rats

**Fig. 1:**
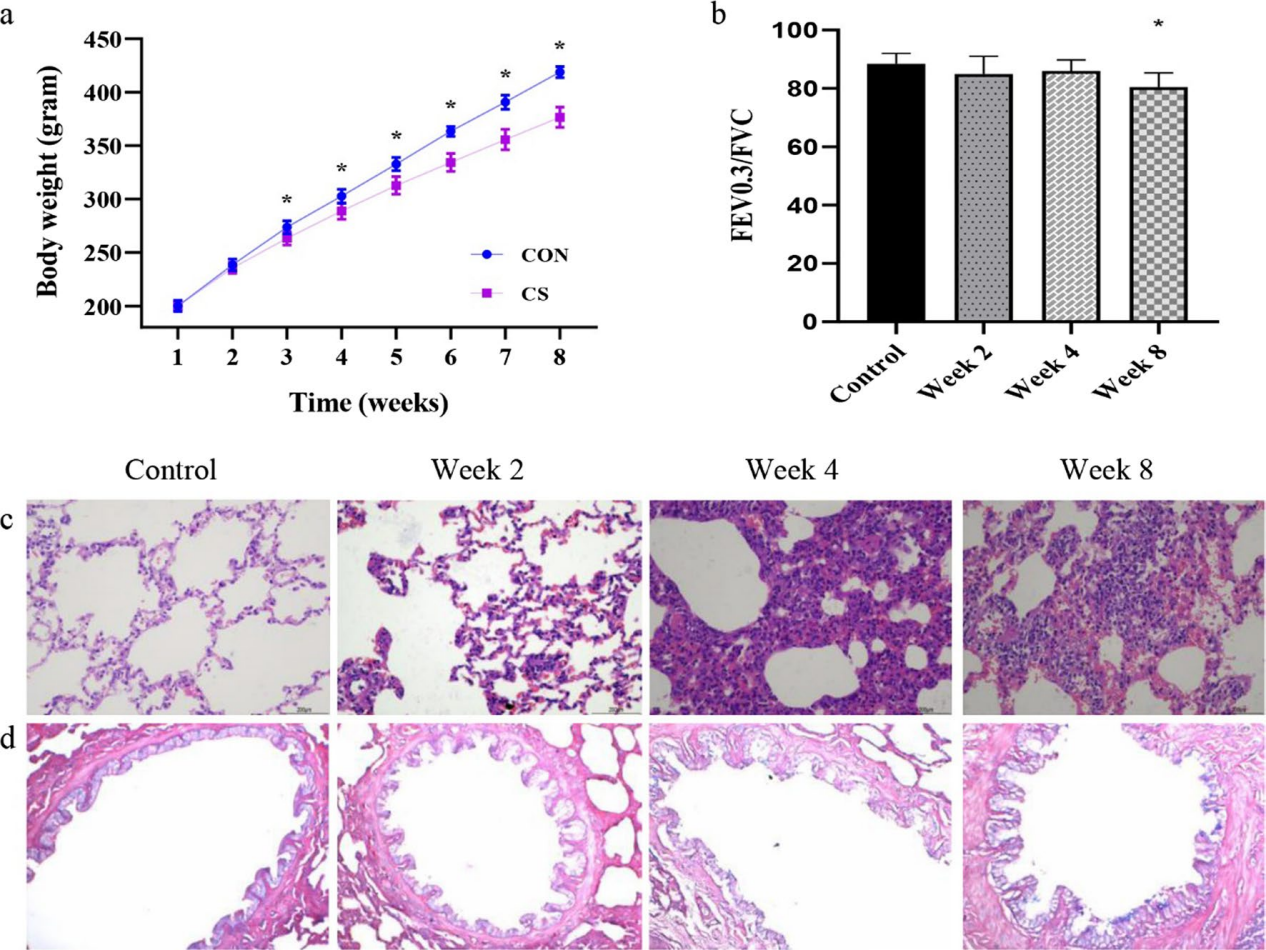
Clinical characterization of the cigarette smoke-induced COPD rat model. **a** Body weight changes in the CS-induced COPD rat model (n = 12, *p < 0.01); **b** Pulmonary function in rats (n = 10, *p < 0.01); **d** HE staining of alveolar tissue at an original magnification 200 × ; **d** AB-PAS staining for mucus expression in the epithelium of the bronchus at an original magnification 200 ×

There was no difference in the baseline body-weight between the two groups. However, the body weight of the CS group was reduced compared to the control group after CS exposure for 3 weeks, (*p* < 0.01, Fig. 1a). FEV0.3/FVC were significantly lower in the CS group than that in the control group on week 8, indicating CS exposure caused obvious airflow obstruction compared with room-air exposure controls (*p* < 0.01, Fig. 1b).

The H&E staining showed bronchial epithelial detachment and expansion and rupture of the alveolar space after CS exposure for 4 weeks. The rat lung bronchial epithelial cells were denatured, adhered, and partially detached and alveolar wall thinning occurred, while the alveolar space expanded, ruptured or had bullae formed in it after 8 weeks CS exposure (Fig. 1c). AB-PAS staining showed bronchial epithelial goblet cells in the rats became larger after 4 weeks CS exposure. And the number of goblet cells (in blue) in the bronchial epithelium was dramatically elevated and the size of goblet cells was enlarged with hypertrophy and hypersecretion after 8 weeks CS exposure (Fig. 1D).

### Dynamic urinary proteome changes in CS-induced COPD rats

**Fig. 2:**
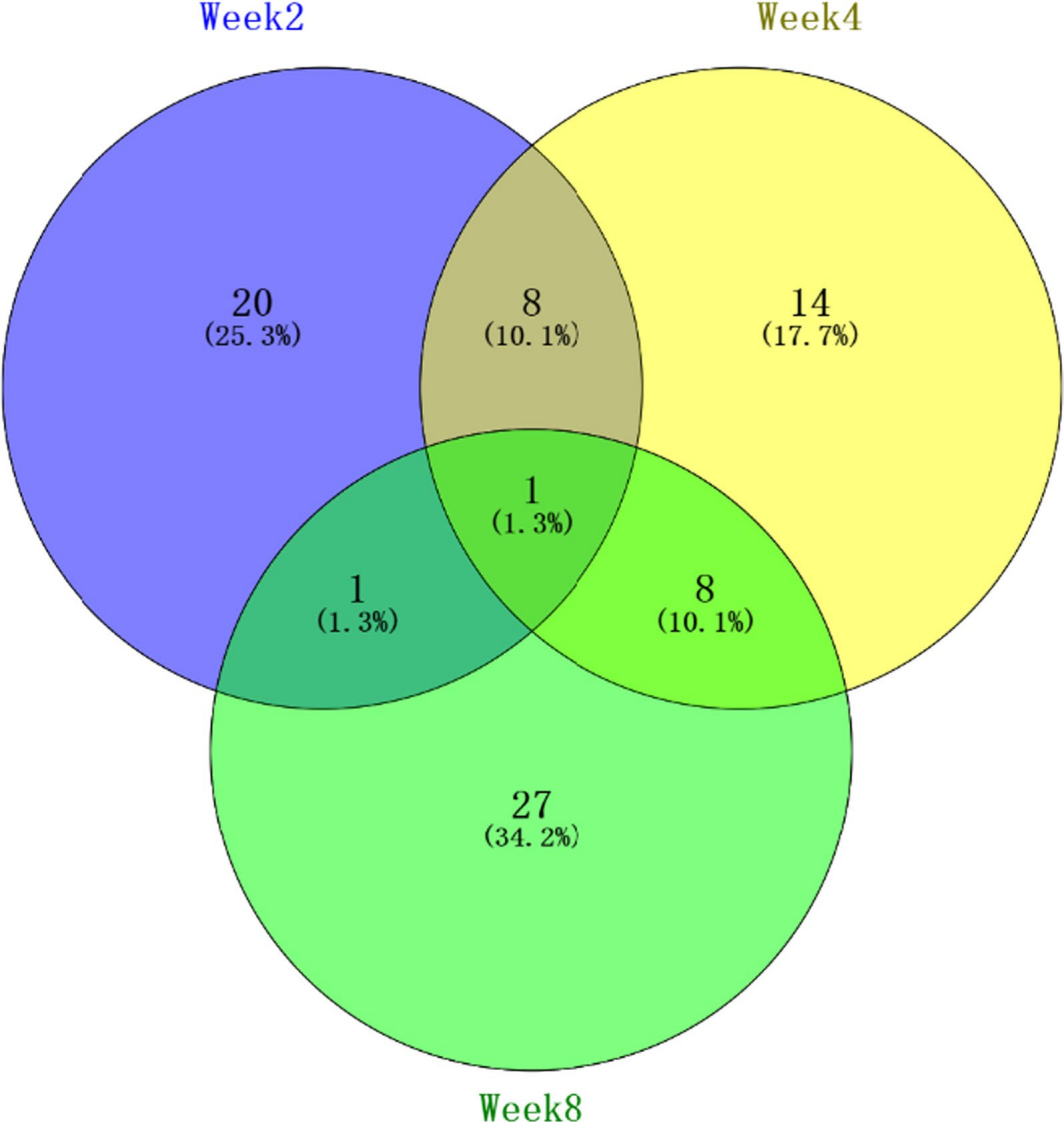
The Venn diagram of the differential urinary proteins at week 2, 4 and 8 in cigarette smoking-induced COPD rat model

After LC–MS/MS analysis, 340 urinary proteins were identified with at least 2 unique peptides (FDR 1%). All identification and quantitation details are listed in Additional file 1: Table S1. Among these, 79 proteins were significantly changed (fold change-1.5, *p* < 0.05), and 56 proteins had human orthologs. There were 30, 31, and 37 differential proteins after CS exposure for 2, 4 and 8 weeks, respectively, (Table 1). The overlap of the differential proteins identified at different COPD stages is shown as a Venn diagram (Fig. 2).

**Table 1:** Dynamic urinary proteome changes in the cigarette smoke-induced COPD rats

Accession	Protein names	week 2	week 4	week 8	Human orthologs
P23764	Glutathione peroxidase 3	3.68	2.14	1.75	P22352
P01015	Angiotensinogen	2.40	1.94		None
P02761	Major urinary protein	2.39	2.52		None
Q9WUW9	Sulfotransferase 1C2A	2.20	1.62		O00338
P29598	Urokinase-type plasminogen activator	1.98	1.70		P00749
P17475	Alpha-1-antiproteinase	1.81	1.60		P01009
P48199	C-reactive protein	1.73	1.72		P02741
P07151	Beta-2-microglobulin	1.65	1.80		P61769
Q99PS8	Histidine-rich glycoprotein	0.41	0.20		None
P22282	Cystatin-related protein 1		0.41	3.61	None
P02780	Secretoglobin family 2A member 2		0.47	3.36	None
P22283	Cystatin-related protein 2		0.47	3.36	None
Q9JHB9	Secretoglobin family 2A member 1		0.48	3.11	None
P02782	Prostatic steroid-binding protein C1		0.44	3.09	None
P08592	Amyloid-beta precursor protein		0.57	0.61	P05067
P01681	Ig kappa chain V region S211		3.57	0.38	None
Q66H12	Alpha-N-acetylgalactosaminidase		2.50	0.26	P17050
P00758	Kallikrein-1	1.56		0.55	None
P14841	Cystatin-C	3.70			P01034
P50430	Arylsulfatase B	3.61			P15848
Q63621	Interleukin-1 receptor accessory protein	2.47			Q9NPH3
Q68FP1	Gelsolin	2.01			P06396
P31211	Corticosteroid-binding globulin	1.90			None
Q9Z0W7	Chloride intracellular channel protein 4	1.88			Q9Y696
Q01177	Plasminogen	1.80			P00747
Q9JJ40	Na (+)/H (+) exchange regulatory cofactor NHE-RF3	1.68			Q5T2W1
P01048	T-kininogen 1	1.68			None
Q03626	Murinoglobulin-1	1.65			None
Q64240	Protein AMBP	1.61			P02760
P36953	Afamin	1.60			P43652
P20611	Lysosomal acid phosphatase	1.59			P11117
Q9QX79	Fetuin-B	1.59			None
Q642A7	Protein FAM151A	1.58			Q8WW52
P02651	Apolipoprotein A-IV	1.57			None
Q920A6	Retinoid-inducible serine carboxypeptidase	1.51			Q9HB40
P13432	SMR1 protein	0.64			None
P42854	Regenerating islet-derived protein 3-gamma	0.32			Q06141
P29534	Vascular cell adhesion protein 1	0.19			P19320
B5DFC9	Nidogen-2		0.44		Q14112
O35217	Multiple inositol polyphosphate phosphatase 1		1.90		Q9UNW1
O35763	Moesin		1.68		P26038
P00762	Anionic trypsin-1		4.66		P07478
P01836	Ig kappa chain C region, A allele		0.66		None
P04937	Fibronectin		1.81		P02751
P08649	Complement C4		0.43		P0C0L4
P24090	Alpha-2-HS-glycoprotein		0.39		None
P48500	Triosephosphate isomerase		0.48		P60174
Q4QQW8	Putative phospholipase B-like 2		2.65		Q8NHP8
Q5M876	N-acyl-aromatic-l-amino acid amidohydrolase		1.78		Q96HD9
Q64230	Meprin A subunit alpha		1.69		Q16819
Q6P7A9	Lysosomal alpha-glucosidase		0.47		P10253
Q80W57	Broad substrate specificity ATP-binding cassette transporter ABCG2		2.41		Q9UNQ0
P01835	Ig kappa chain C region, B allele			0.54	P0DOX7
P02781	Prostatic steroid-binding protein C2			4.03	None
P06866	Haptoglobin			2.54	P00739
P06882	Thyroglobulin			0.37	P01266
P11951	Cytochrome c oxidase subunit 6C-2			0.61	P09669
P15978	Class I histocompatibility antigen, Non-RT1.A alpha-1 chain			2.80	P01889
P16391	RT1 class I histocompatibility antigen, AA alpha chain			0.46	P04439
P25236	Selenoprotein P			0.22	P49908
P28826	Meprin A subunit beta			0.58	Q16820
P31044	Phosphatidylethanolamine-binding protein 1			2.69	P30086
P36374	Prostatic glandular kallikrein-6			1.58	None
P36376	Glandular kallikrein-12, submandibular/renal			1.78	None
P97546	Neuroplastin			0.63	Q9Y639
Q09030	Trefoil factor 2			0.02	Q03403
Q5FVH2	5′-3′ exonuclease PLD3			0.58	Q8IV08
Q5U2Q3	Ester hydrolase C11orf54 homolog			0.31	Q9H0W9
Q62839	Golgin subfamily A member 2			2.73	Q08379
Q63678	Zinc-alpha-2-glycoprotein			2.68	None
Q6AYR9	Tetraspanin-1			3.48	O60635
Q6AYS7	Aminoacylase-1A			0.44	Q03154
Q6IRK9	Carboxypeptidase Q			0.62	Q9Y646
Q6TUD4	Protein YIPF3			0.60	Q9GZM5
Q8VD89	Ribonuclease pancreatic gamma-type			5.01	None
Q99J86	Attractin			3.02	O75882
Q9JHY1	Junctional adhesion molecule A			0.57	Q9Y624
Q9JI85	Nucleobindin-2			2.59	P80303
H1UBN0	Copine-7			0.18	None

### Gene ontology (GO) analysis of the differential proteins

**Fig. 3:**
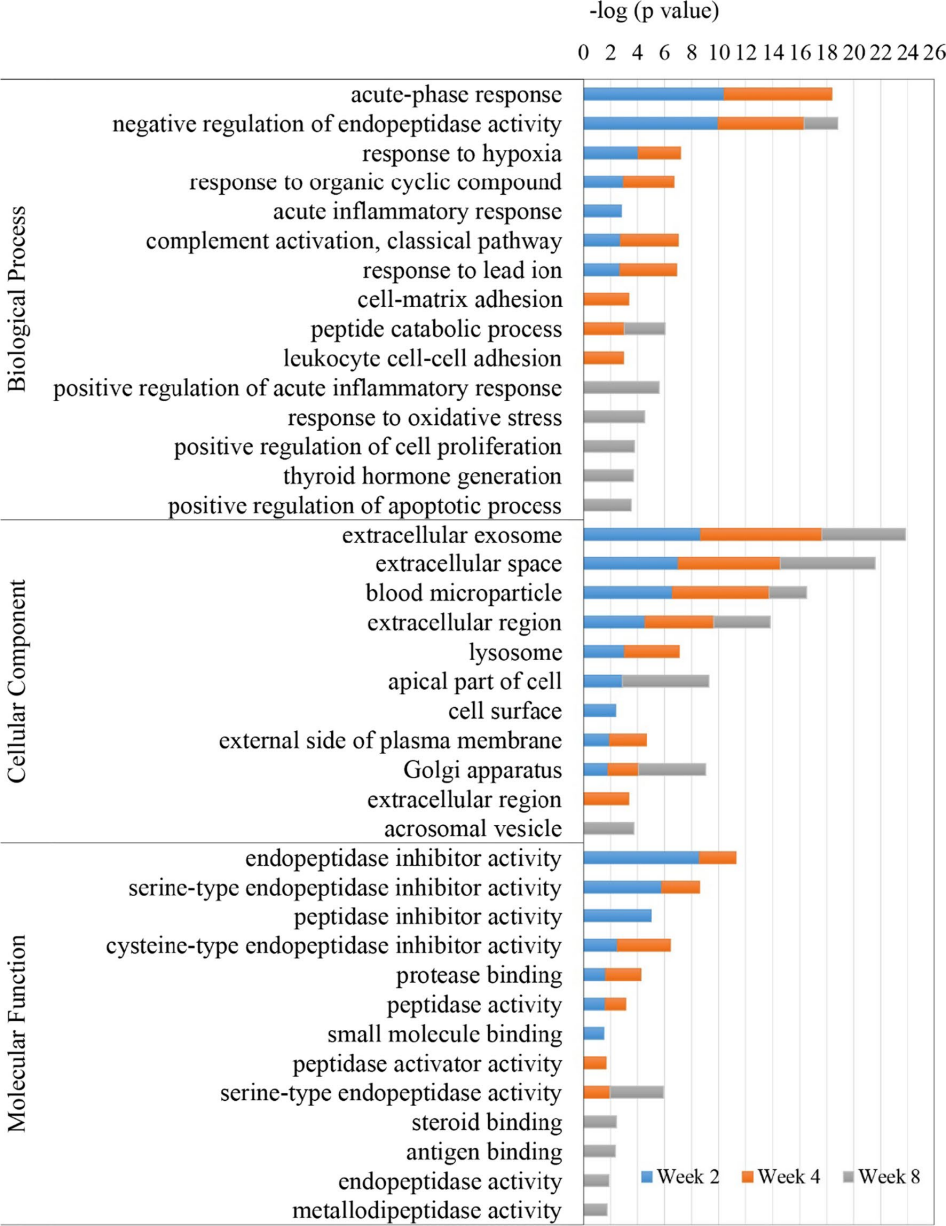
GO enrichment analysis of the differential urinary proteins at week 2, 4 and 8 in cigarette smoke-induced COPD rat model

GO enrichment analysis was performed on the 79 differential urinary proteins in CS-induced COPD rats. GO revealed that these differential proteins were involved in the regulation of a host of biological processes (Fig. 3). Five biological processes were enriched at week 2 and 4 only, namely acute-phase response, response to organic cyclic compound, complement activation classical pathway, and response to lead ion. Another five biological processes were only enriched at week 8, namely positive regulation of acute inflammatory response, response to oxidative stress, positive regulation of cell proliferation, thyroid hormone generation, and positive regulation of the apoptotic process.

Most of the differential urinary proteins were associated with extracellular exosomes, extracellular space, blood microparticle, and extracellular region among the cellular components (Fig. 3). In the molecular function category, endopeptidase inhibitor activity, serine-type endopeptidase inhibitor activity, cysteine-type endopeptidase inhibitor activity, protease binding, and peptidase activity were overrepresented at week 2 and 4. The steroid binding, antigen binding, endopeptidase activity, and metallodipeptidase activity were overrepresented at week 8 (Fig. 3). These results indicate that the urine proteome can reflect biological responses in the body during the progression of CS exposure.

### Protein–protein interaction (PPI) network of the differential proteins

**Fig. 4:**
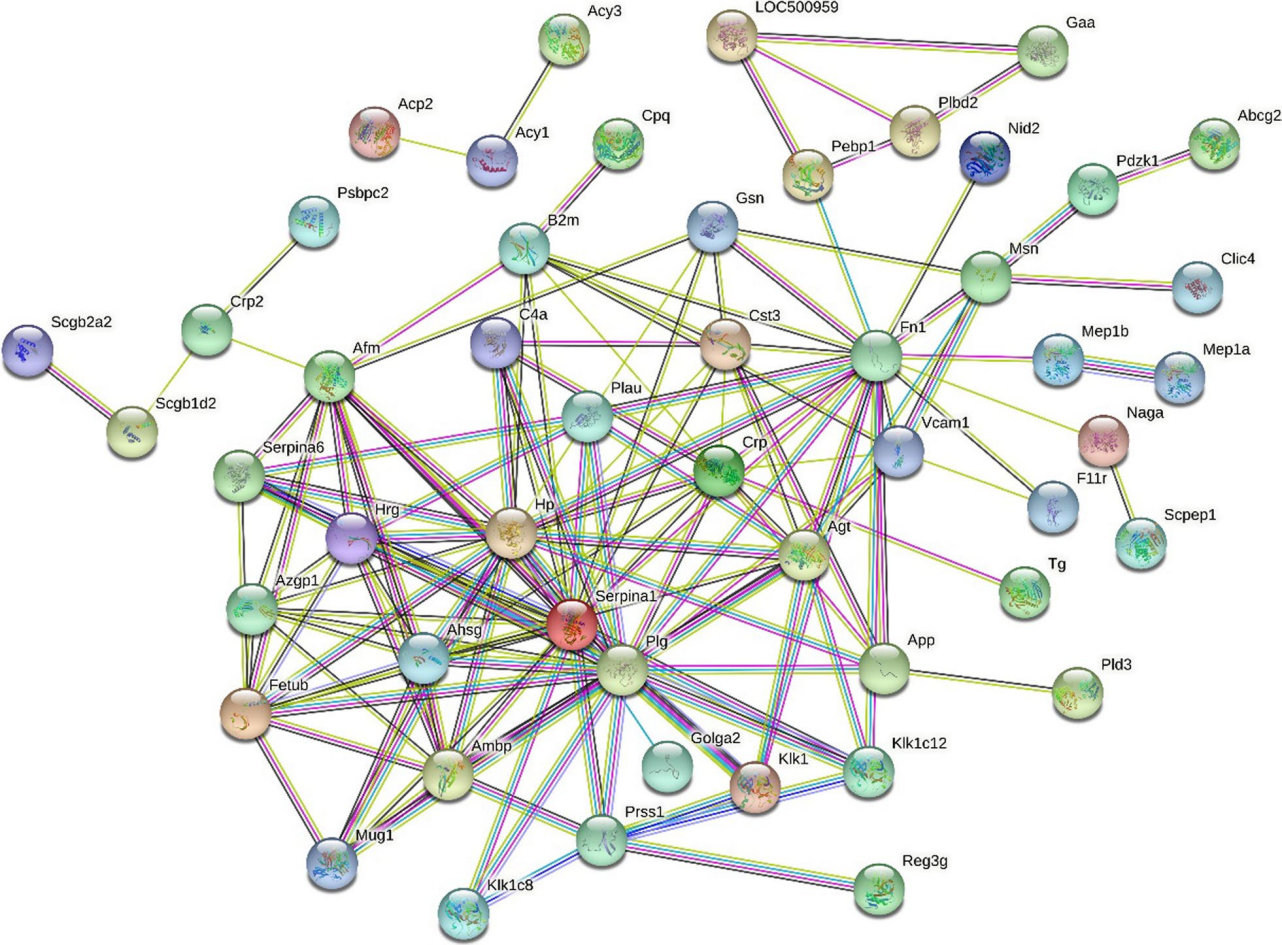
STRING PPI network analysis of the differential urinary proteins in cigarette smoke-induced COPD rat model. The number of nodes is 68, the average node degree is 4.29, and the average local clustering coefficient is 0.491 (*p-value* < 1.0e–16)

To better understand the pathogenic mechanisms in COPD, the PPI network of the 79 differential proteins was constructed by STRING (Fig. 4). The number of included nodes was 68, the details were listed in Additional file 1: Table S2. The average node degree is 4.29, and the average local clustering coefficient is 0.491 (*p* < 1.0e–16). The results revealed that the differential urinary proteins had more intra- and intermolecular interactions than expected for a random set of proteins of similar size, drawn from the genome. Such an enrichment indicates that the differential proteins are closely biologically connected as a group.

### Urinary candidate biomarker for CS-induced COPD

To find more reliable urinary differential proteins associated with CS-induced COPD, the twenty remaining urine samples were validated by LC–MS/MS. Thirteen urinary proteins with human orthologs were verified as potential biomarkers of CS-induced COPD (Table 2). Of these 13 candidate biomarkers, eight proteins have been reported as biomarkers of certain diseases. In addition, four proteins are known to be associated with COPD, namely urokinase-type plasminogen activator, plasminogen, fibronectin, and trefoil factor 2. At week 2 and 4, seven differential proteins were verified as early screening biomarkers of CS-induced COPD, when no obvious changes in pulmonary function or pathological morphology were observed. At week 8, six differential proteins were verified as diagnostic biomarkers of CS-induced COPD when obvious airflow obstruction compared with room-air exposure controls was detected.

**Table 2:** Potential urinary protein biomarkers for the cigarette smoke-induced COPD

Accession	Protein names	Human orthologs	Trend	Biomarkes	COPD-related
Q9WUW9	Sulfotransferase 1C2A	O00338	↑		
P29598	Urokinase-type plasminogen activator	P00749	↑	Lung adenocarcinoma [30, 31]	Yes
Q01177	Plasminogen	P00747	↑	Hepatocellular carcinoma [32], Acute-on-chronic liver failure [33]	Yes
P04937	Fibronectin	P02751	↑	COPD [18], Duchenne muscular dystrophy [34]	Yes
Q920A6	Retinoid-inducible serine carboxypeptidase	Q9HB40	↑		
P08592	Amyloid-beta precursor protein	P05067	↓		
B5DFC9	Nidogen-2	Q14112	↓	Ovarian cancer [35]	
P31044	Phosphatidylethanolamine-binding protein 1	P30086	↑		
Q9JI85	Nucleobindin-2	P80303	↑	Breast cancer [36]	
P19804	Nucleoside diphosphate kinase B	P22392	↑		
Q6AYR9	Tetraspanin-1	O60635	↑	Colon cancer [37], Acute rejection in kidney transplantation [38]	
Q09030	Trefoil factor 2	Q03403	↓	Pancreatic cancer [39], Precancerous lesion [40], COPD [26, 27]	Yes
Q9JHY1	Junctional adhesion molecule A	Q9Y624	↓	Multiple myeloma [41], Glioma [42]	

## Discussion

COPD is a prevalent respiratory disease showing an annual increase in morbidity and mortality rates. Given the prevalence and negative impact of comorbidities in individuals with COPD, early screening and detection that leads to meaningful interventions may improve patients’ outcomes and quality of life. However, early COPD diagnosis has remained challenging due to small impact of early lung function loss. In this study, we aimed to explore potential urinary biomarkers for CS-induced COPD. Overall, we systematically investigated dynamic changes in urinary proteome in a CS-induced COPD rat model for the first time based on proteomics analysis. A total of 340 urinary proteins were identified, of which 79 were significantly changed (30, 31, and 37 at week 2, 4 and 8, respectively). And 13 urinary proteins with human orthologs were verified as potential biomarkers for CS-induced COPD (Table 2). Of these 13 candidate biomarkers, eight proteins have been reported as biomarkers of certain diseases, and four proteins are known to be associated with COPD.

At week 2 and 4, seven differential proteins were verified as early screening biomarkers of CS-induced COPD, when no obvious changes in pulmonary function and pathological morphology were observed. Of these seven early screening biomarkers, Fn has been reported as a serum biomarker of COPD [7, 18]. Fn is a high molecular weight glycoprotein that is present in the body as two major isomers: a soluble circulating form and an insoluble extracellular matrix isomer [19]. Although serum Fn has many functions, its primary role is to promote wound repair following injury or infection by mediating cellular adhesion, motility, differentiation, apoptosis and hemostasis [20]. Using immunohistochemical analysis, the expression of Fn in bronchial vessels has been negatively correlated with FEV1 values in patients with COPD [21]. Moreover, in a study of 4787 subjects with mild-to-moderate COPD, Man et al. observed that the circulating Fn to CRP ratio was independently associated with all-cause mortality of the COPD patients at more than 7 years follow-up [18]. In the current study, the urinary Fn content increased nearly twofold in 2 weeks in CS exposed rats, indicating that urinary Fn may be a promising biomarker for early screening of COPD.

In addition, Plg and uPA have also been implicated in the progression of COPD [22]. The plasminogen activator system, including Plg, uPA, tPA and PAI-1, have diverse functions related to the inflammatory response in mammals [23]. Following injury, Plg extravasates into lung tissue, and cleavage of Plg to plasmin by uPA, stimulates inflammatory and epithelial cell cytokine production and mesenchymal cell proliferation. Immunohistochemical staining analysis has revealed marked elevation of uPA expression in the small airway epithelia of COPD patients by [24]. According to an in vitro study, upregulation of uPA expression might modulate the small airway remodeling in COPD by promoting epithelial-mesenchymal transition [25]. Our previous study revealed that urinary Plg increased 1.6-fold in OVA-induced asthma mice compared to controls [13]. In the current study, the expressions of uPA and Plg in the urinary proteome were both up-regulated nearly twofold in the CS group at week 2 and 4.

At week 8, six differential proteins were verified as diagnostic biomarkers of CS-induced COPD, when obvious airflow obstruction compared with room-air exposure controls was detected. Of these six diagnostic biomarkers, TFF2 has been reported as a serum and a bronchioalveolar lavage fluid (BAL) biomarker of COPD [26, 27]. TFFs 1, 2 and 3 are co-secreted with mucin throughout the body and proposed to be involved in tissue regeneration, proliferation and protection [28]. Mounting evidence shows that TFFs have therapeutic potential in lung disease, and TFF2 promoted repair and had anti-apoptotic effects on epithelial cells in the lung [29]. Viby et al. reported a fourfold increase of TFF2 in serum samples from COPD patients; however, a similar increase was not detected in the sputum [26]. Subsequently, they reported an increased level of TFF2 in BAL fluids from COPD patients and a positive correlation between the level of TFF2 and lung function [27]. Interestingly, we found that the urinary concentration of TFF2 was extremely down-regulated in CS-induced COPD rats, which suggests that the excretion of TFF2 is reduced upon the CS exposure. Taken together, we speculated that the exogenous delivery of TFF2 may prevent the progression of COPD, but this hypothesis needs further investigation.

Furthermore, we compared the urinary differential protein profile of CS-induced COPD with those of OVA-induced asthma [13]. We only identified five differential proteins shared by these two disease states, and these proteins shared a common change trend. The five differential proteins are alpha-1-antiproteinase, beta-2-microglobulin, plasminogen, protein AMBP and haptoglobin. This may be because OVA-induced asthma is a more TH2 based eosinophilic inflammation, while in the CS-induced COPD model is more TH1 based inflammation. Of the 13 candidate biomarkers for CS-induced COPD, eight proteins have been reported as biomarkers of certain diseases (Table 2). And most of these diseases were mainly Th2 response, such as lung adenocarcinoma, hepatocellular carcinoma, ovarian cancer, breast cancer, colon cancer, pancreatic cancer. Taken together, the urinary proteome could be a promising resource for studies of COPD biomarkers.

As a preliminary study, we found candidate urinary biomarkers associated with the development of COPD based on a CS-exposed rat model. Variables that impact clinical samples, such as medication, surgery, and patients’ living habits, were excluded. In the future, proteomics studies in large derivation, along with validation cohorts of patients with well-phenotype COPD and other obstructive lung diseases, may be required for the translation of urinary biomarkers into clinical settings. Furthermore, a combined panel of biomarkers capturing different pathways related to COPD pathophysiology may be required for use in clinical practice.

## Conclusions

In conclusion, we investigated dynamic changes of urinary proteome in CS-induced COPD rat model. Our results reveal that urinary proteome could sensitively reflect pathophysiological changes in the development of COPD, and may advance the knowledge of pathogenesis of COPD in CS exposure. More important, we identified candidate urinary biomarkers which may be utilized for early screening, diagnosis and/or prognosis of COPD.

## Supplementary Information

**Additional file 1: Table S1.** All identification and quantitation details in COPD rats. **Table S2.** The details of the nodes in PPI.

## Data Availability

The datasets used and/or analyzed during the current study are available from the corresponding author on reasonable request.

## Abbreviations

BAL: Bronchioalveolar lavage fluid
COPD: Chronic obstructive pulmonary disease
CS: Cigarette smoking
Fn: Fibronectin
LC–MS/MS: Liquid chromatography coupled with tandem mass spectrometry
PPI: Protein–protein interaction
Plg: Plasminogen
uPA: Urokinase-type plasminogen activator
TFF2: Trefoil factor 2
